# Editorial: Infectious and inflammatory mechanistic underpinnings of CFS/ME

**DOI:** 10.3389/fneur.2023.1082473

**Published:** 2023-01-19

**Authors:** Alexander Robert Burton

**Affiliations:** ^1^Neuroscience Research Australia, Sydney, NSW, Australia; ^2^School of Medicine, Western Sydney University, Campbelltown, NSW, Australia; ^3^School of Medical Sciences, University of New South Wales, Randwick, NSW, Australia

**Keywords:** Chronic Fatigue Syndrome/Myalgic Encephalomyelitis (CFS/ME), mechanisms, homeostasis, treatment, inflammation

Chronic Fatigue Syndrome/Myalgic Encephalomyelitis (CFS/ME) is a debilitating disorder characterized by medically unexplained, incapacitating fatigue as well as constitutional and neuropsychiatric symptoms of at least 6 months duration (1, 2). While the actual specific mechanisms behind CFS/ME remain obscure, it has been long known that CFS/ME could be triggered by viral infections: fatigue symptoms were associated with the polio outbreaks around the 1930s to the 1950s and other later outbreaks (3). Indeed, one of the earliest mentions of the modern term “Chronic Fatigue Syndrome” was derived from the “Chronic Epstein-Barr virus syndrome” back in 1988 (4).

At that time, researchers and medical professionals were deliberating whether the condition may largely be psychosomatic, or whether the debilitating fatigue in fact had a physiological basis (3, 5, 6). To date, there are two main impediments that hamper elucidation of possible physiological/pathological mechanisms behind CFS/ME: the heterogeneity of the condition, and the lack of objective biomarkers that would enable a definitive diagnosis. As a result of this, CFS/ME has been frequently relegated into the “too hard basket” by clinicians and researchers, resulting in stigmatization of sufferers who were often classified as having “psychosocial” problems (7, 8).

In order to reduce the effect of heterogeneity, ambitious large longitudinal cohort studies such as the Dubbo Infectious Outcomes Study (DIOS) were conducted (9). Variables were reduced by studying homogeneous cohorts of people who experienced post-infective fatigue following documented viral infections. In spite of this careful methodology and longitudinal follow-up until complete recovery, or until, at 6 months, a diagnosis of CFS could be made, no definitive immunological/inflammatory abnormality could be identified as a marker of post-infective fatigue (9, 10). Nevertheless, the DIOS cohorts lead to an interesting insight. It was found that the severity of the *initial* infection strongly predicted the duration and severity of the post infective illness and fatigue. More specifically, genetically determined variations in the intensity of the inflammatory response to the initial infection predicted the severity of the acute sickness response and forecasted the recovery time following infection (11, 12).

Recent reanalysis of the DIOS cohort data focusing on genetic changes—including some associated with autoimmunity such as the inflammasome, IL6, IL1beta, IL10, and others—reaffirmed these earlier findings and findings are presented in this special edition of Frontiers in Neurology in “Predictors of chronic fatigue syndrome and mood disturbance after acute infection.” To put it simply: the severity of the initial insult or shock to the body's homeostasis is the best predictor of whether an individual goes on to develop CFS/ME. In the mini-review “Animal models for neuroinflammation and potential treatment methods,” Tamura et al., states the onset of CFS/ME in animals is often associated with neuroinflammation which follows either bacterial or viral infections and it is the degree of neuroinflammation that correlates with the severity of several CFS/ME symptoms.

With the recent rise in reports of long COVID associated with the SARS-Cov2 virus and its variants, there has been a renewed interest in the effects of post-viral fatigue and CFS/ME. A brief search of the terms “long covid” AND “fatigue” in the Scopus database yields over a thousand publications since the beginning of the 2019 pandemic. A growing body of evidence suggests long COVID seemingly mimics CFS/ME (13, 14): Postural Orthostatic Syndrome (POTS) is commonly reported in both conditions (15). However, long COVID also displays a myriad of multi-organ system symptoms such as dyspnoea which is rarely seen in CFS/ME, and the lingering veiled effects of the SARS-Cov2 virus seemingly add further layers of heterogeneous complexity to long COVID (15–17). In this edition Tate et al., suggest that while there is still considerable symptomatic overlap in both ongoing conditions, there is also unique signaling in neuroinflammatory mechanisms caused by the initial SARS-CoV-2 infection (resulting in long COVID) vs. those involved in other cases of CFS/ME. However, the overlapping symptomatology tentatively supports the concept of a similar dysfunctional CNS component collectively shared in CFS/ME and long COVID that is controlling and sustaining the pathophysiological state (“Molecular mechanisms of neuroinflammation in ME/CFS and Long COVID to sustain disease and promote relapses.”).

To date, the mechanisms triggering and perpetuating CFS/ME still remain a mystery. As if the complexity of understanding the heterogenous nature of CFS/ME was not difficult enough, long COVID takes it to a more perplexing level. Hence, the challenge of understanding long COVID remains even greater: “*Like long COVID, ME/CFS is an intractable, heterogeneous condition. Its causes are unclear, preventing long-term effective treatment. The urgent need for high-quality, imaginative and ambitious research should therefore not be undermined by downplaying the current impact of this condition on millions of people around the world* (18)*.”* SARS-Cov2 is relatively new and understanding of its mechanisms and effects will take time. Long COVID and CFS/ME may indeed share a number of commonalities beyond the fatigue, however the two conditions are not interchangeable. In the broadest sense, both conditions are the result of a significant virological/immunological stressors which leads to ongoing and debilitating fatigue.

It can be speculated: “*…that post-infective CFS may be understood as a physiological variant of posttraumatic stress syndrome. Here, the stressor of a severe infection in vulnerable individuals may trigger alterations in neurophysiological systems that perpetuate symptoms of the acute infection, leading to the development of CFS”* (19). This broad hypothesis encompasses a holistic view of the myriad of stressors or straws which may eventually break the proverbial camel's back, disrupting homeostasis and leading to a state of physiological hyper-responsivity to future stressors. Vollmer-Conna concludes: “*…it seems that to attain a true understanding of this enigmatic disorder, we must be able to bridge the psychology/medicine divide. The full answer is not likely to be contained in a single abnormality or change at the molecular level but has to take into consideration that major regulatory systems interact dynamically to maintain homeostasis and body integrity”* (19).

Decades later, and despite substantial hypothesis-driven research, the pathophysiology of CFS/ME remains enigmatic, and no curative treatment exists. This special edition of Frontiers in Neurology renews a focus on this poorly understood condition by offering innovative discussions of several potential pathophysiological mechanisms, mechanistic hypotheses and potential avenues for future treatments.

## Author contributions

The author confirms being the sole contributor of this work and has approved it for publication.

## References

[1] Fukuda K Straus SE Hickie I Sharpe MC Dobbins JG Komaroff A. The chronic fatigue syndrome: a comprehensive approach to its definition and study. International Chronic Fatigue Syndrome Study Group. Ann Intern Med. (1994) 121:953–9. 10.7326/0003-4819-121-12-199412150-00009 7978722

[2] Carruthers BM Jain AK De Meirleir KL Peterson DL Klimas NG. Myalgic encephalomyelitis/chronic fatigue syndrome: clinical working case definition, diagnostic and treatment protocols. J Chronic Fatigue Syndr. (2003) 11:7–115. 10.1300/J092v11n01_02

[3] Jenkins R. Epidemiology: lessons from the past. Br Med Bull. (1991) 47:952–65. 10.1093/oxfordjournals.bmb.a072523 1794093

[4] Holmes GP Kaplan JE Gantz NM Komaroff AL Schonberger LB Straus SE. Chronic fatigue syndrome: a working case definition. Ann Intern Med. (1988) 108:387–9. 10.7326/0003-4819-108-3-387 2829679

[5] Kroenke K Birrer RB. Chronic fatigue syndrome: is it real? Postgrad Med. (1991) 89:44–55. 10.1080/00325481.1991.11700814 1990396

[6] Wallace PG. Post-viral fatigue syndrome. Epidemiology: a critical review. Br Med Bull. (1991) 47:942–51. 10.1093/oxfordjournals.bmb.a072522 1794092

[7] Afari N Buchwald D. Chronic fatigue syndrome: a review. Am J Psychiatry. (2003) 160:221–36. 10.1176/appi.ajp.160.2.221 12562565

[8] Fong K. Myalgic Encephalomyelitis/Chronic Fatigue Syndrome Advisory Committee: Report to the NHMRC Chief Executive Officer. Canberra, ACT: National Health and Medical Research Council (2019).

[9] Hickie I Davenport T Wakefield D Vollmer-Conna U Cameron B Vernon SD. Dubbo Infection Outcomes Study Group. Post-infective and chronic fatigue syndromes precipitated by viral and non-viral pathogens: prospective cohort study. BMJ. (2006) 333:575. 10.1136/bmj.38933.585764.AE 16950834 PMC1569956

[10] Vollmer-Conna U Cameron B Hadzi-Pavlovic D Singletary K Davenport T Vernon S. Postinfective fatigue syndrome is not associated with altered cytokine production. Clin Infect Dis. (2007) 45:732–5. 10.1086/520990 17712757

[11] Vollmer-Conna U Piraino BF Cameron B Davenport T Hickie I Wakefield D. Dubbo Infection Outcomes Study Group. Cytokine polymorphisms have a synergistic effect on severity of the acute sickness response to infection. Clin Infect Dis. (2008) 47:1418–25. 10.1086/592967 18937577

[12] Piraino B Vollmer-Conna U Lloyd AR. Genetic associations of fatigue and other symptom domains of the acute sickness response to infection. Brain Behav Immun. (2012) 26:552–8. 10.1016/j.bbi.2011.12.009 22227623 PMC7127134

[13] Wostyn P. COVID-19 and chronic fatigue syndrome: Is the worst yet to come? Med Hypotheses. (2021) 146. 10.1016/j.mehy.2020.110469 33401106 PMC7836544

[14] Komaroff AL Lipkin WI. Insights from myalgic encephalomyelitis/chronic fatigue syndrome may help unravel the pathogenesis of postacute COVID-19 syndrome. Trends Mol Med. (2021) 27:895–906. 10.1016/j.molmed.2021.06.002 34175230 PMC8180841

[15] Yong SJ. Long COVID or post-COVID-19 syndrome: putative pathophysiology, risk factors, and treatments. Infect Dis. (2021) 53:737–54. 10.1080/23744235.2021.1924397 34024217 PMC8146298

[16] Datta SD Talwar A Lee JT. A proposed framework and timeline of the spectrum of disease due to SARS-CoV-2 infection: illness beyond acute infection and public health implications. JAMA. (2020) 324:2251–2. 10.1001/jama.2020.22717 33206133

[17] Amenta EM Spallone A Rodriguez-Barradas MC El Sahly HM Atmar RL Kulkarni PA. Postacute COVID-19: an overview and approach to classification. Open Forum Infect Dis. (2020) 7:ofaa509. 10.1093/ofid/ofaa509 33403218 PMC7665635

[18] White P. Long COVID: don't consign ME/CFS to history. Nature. (2020) 587:197–197. 10.1038/d41586-020-03136-0 33173222

[19] Vollmer-Conna U. Chronic fatigue syndrome in adolescence: where to from here? Arch Pediatr Adolesc Med. (2010) 164:880–1. 10.1001/archpediatrics.2010.149 20819973

